# A Hypothesis About Parallelism vs. Seriality in Dreams

**DOI:** 10.3389/fpsyg.2019.02299

**Published:** 2019-10-10

**Authors:** Umberto Barcaro, Paolo Paradisi, Laura Sebastiani

**Affiliations:** ^1^ISTI-CNR, Institute of Information Science and Technologies “A. Faedo”, Pisa, Italy; ^2^Basque Center for Applied Mathematics, Bilbao, Spain; ^3^Department of Translational Research and New Technologies in Medicine and Surgery, University of Pisa, Pisa, Italy

**Keywords:** dream sources, network properties of dream sources, dream experience, parallelism vs. seriality in physiological systems, dream building, complexity, self-organization

The process of dream building implies the construction of a complex network of closely interrelated sources. On the other hand, the dream experience develops as a succession of events. In this paper a hypothesis is advanced about how the psychophysiological system of dream building, which is distributed, acts to provide a serial output. This hypothesis is basically connected with the property, enjoyed by the dream experience, of simultaneously representing a plurality of meanings.

Most of the products of our brain, which consists in an enormous number of cells and of interconnections among them, give serial outputs. In fact, physiological and psychophysiological systems generally interact serially with the environment, as is obvious with regard to, e.g., locomotion, reaching and grasping, and language. Two trivial examples are walking (we move a leg after the other) and speaking (we pronounce a word after the other). Seriality also appears to characterize the control of logical reasoning and, very generally, the flow of consciousness. In the vast literature about the issue of brain parallelism with serial output, a particularly significant role has been played in the last decade by the hypothesis of a Global Neuronal Workspace (GNW). According to a review by Dehaene and Changeux (2011), the GNW interconnects specialized, automatic and unconscious processors and, at the same time, is able to encode conscious contents by means of the sustained activity of a fraction of its neurons, the others being inhibited. The pattern of parallel computation with serial output is thus closely connected with the theoretical modeling of consciousness. In fact, quoting (Baars et al., 2013),

“the capacity of consciousness at any given moment seems limited to one consistent scene. The flow of such scenes is serial, in contrast with the massive parallelism of the brain as observed directly.”

A number of studies have also dealt with this issue in the framework of complexity theories, thus involving concepts of self-organization and emergent properties in cooperative dynamics (see, e.g., Werner, 2012; Allegrini et al., 2013, 2015; Paradisi et al., 2013; Paradisi and Allegrini, 2017).

As to dreaming, a fundamental property of this activity is that of making connections (see, e.g., Hartmann, 2010). The memory sources of a dream generally refer to recent or remote events in the dreamer's life, including significant “present concerns.” The sources exhibit a network pattern that can qualitatively and quantitatively be described by an appropriate graph representation (Barcaro and Carboncini, 2018). To tackle the issue of how a serial output is obtained as a result of a distributed process, our reflections rely on four phenomena that characterize the network pattern (Barcaro et al., 2016):

1. “Pervasive links” exist, i.e., semantic links among sources that are related to a plurality of sources;
2. A heuristic rule can account for the construction of these links: they are such that the dreamer's present concerns are made less negative or even reversed into positive;
3. After an initially identified present concern, a second more important one can be recognized (this phenomenon has been called “shift of the present concern”);
4. The dream experience fulfills a “representative function”: the overcoming of negative contents is actually represented in the dream.

The first three phenomena are connected with the parallelism of the dream-building system, while the fourth is connected with the seriality of the output.

As to point (2), the heuristic rule closely resembles the Freudian theory of wish fulfillment. However, the literature data that we have considered do not precisely refer to the Freudian Unconscious and do not imply that the rule is valid in all cases.

Our point is that the serial output is created by the system in a conceptually simple way, i.e., by providing a dream plot able to simultaneously represent the overcoming of the present concerns, which are more than one because of the shift of the present concern. This property appears as a typical feature of the dreaming experience.

## Three Examples

To illustrate our point of view we briefly discuss three examples. The rationale for the analysis of dreams consists in observing that (Barcaro et al., 2016):

a) The presence of a non-serial network of dream sources shows that the dream-building system works in a distributed way;
b) More than one present concern is at the basis of the building of the dream;
c) The serial output, i.e., the dream experience, allows the overcoming of the present concerns to be represented simultaneously.

Analyses similar to those carried out for the examples can be performed considering many other dreams that have been reported in the literature, which offers a large amount of dream reports together with associations (see, e.g., Delaney, 1993; Barcaro et al., 2016).

**(i)** The Dream with the house in the courtyard (25-year-old woman, Barcaro et al., 2005):

After being awakened in the morning, the dreamer reported of finding herself inside a house in a courtyard, during a cold night, with other people. After leaving the house they came back and entered another room. Finally, they left the house. In the associations, the other people were immediately identified as her parents and grandparents. The links among the sources provided a closely interconnected pattern, including four pervasive links; for the details of this pattern, we refer to the article from which this example has been taken. The dreamer immediately associated the cold of the night with a feeling of extreme sadness and this feeling with her grandfather's illness. She later indicated a further, very different, present concern, due to her opposition to coming back to the house where they had lived during her adolescence: indeed, her grandfather was the one who most insisted on coming back. Although the feelings related to these two concerns were different and somehow opposite with regard to her grandfather, the serial dream experience managed to represent both feelings, i.e., sadness and desire of definitely leaving that house.

An interesting detail of the dream experience is that, after leaving the house, they entered again and left it again: this well corresponds to the waking-life concern about coming back to the old house.

**(ii)** The Dream with a doctor wearing a white coat (46-year-old woman, Barcaro et al., 2016):

The dreamer reported of being in a hotel room where a doctor with a white coat was visiting her. At the end of the dream, she was skating fast in the room along the spaces among the beds. The associations identified numerous sources and links among sources. The dreamer indicated two present concerns: the wish of improving her relationship with her daughter, and the need to overcome a temporary difficulty in moving. The white coat was the one that her daughter wore in her school lab. The idea of skating was connected with important events in the dreamer's life: her mother had taught her how to skate when she was a child; she had often skated pleasantly together with a partner with whom she had made interesting journeys. Interestingly, the event of skating with that partner was recollected very lately in the course of the associations. The serial dream experience fulfilled a complex representative function: she completely recovered, she was able to relive pleasant experiences of journeys and physical exercise, and she found a tender help on the part of her daughter. The beds in the dream provided a setting able to simultaneously represent a hotel, a medical office, and a skating rink, given by the spaces among the beds.

**Figure 1:**
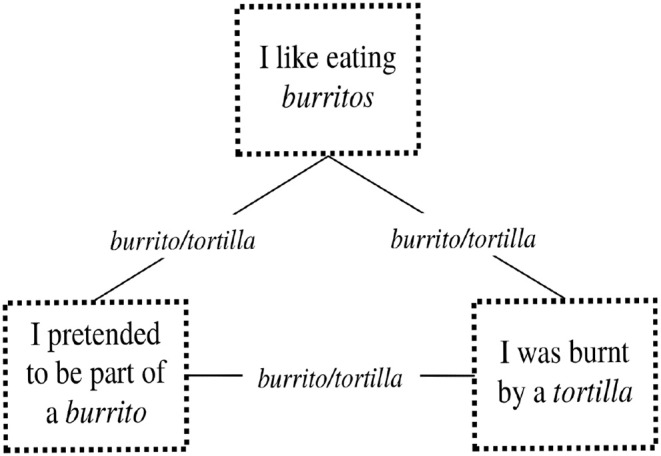
The graph representing the link pattern between the sources of the Dream with the even tortilla is very simple, consisting in a triangle. The nodes are given by the three sources, each pair of which is connected by the pervasive link “burrito/tortilla,” which thus provides the three arcs connecting the nodes.

**(iii)** The Dream with the evil tortilla (2.5-year-old child, Siegel and Bulkeley, 1998):

The small child dreamed of being attacked by an evil tortilla that was trying to smother her. She had always enjoyed eating burritos; recently, she and her older sister had invented the funny play of wrapping themselves inside a blanket, thus pretending to be part of a burrito. On the day before the dream night, the dreamer had suffered a mild burn from touching a tortilla too soon after it had emerged from the toast oven. Thus, the idea of burrito/tortilla provides a pervasive link. Figure 1 shows the graph representation of the link pattern among the sources, which is very simple in this case because of the presence of only one pervasive link. The concern about the burritos possibly being harmful was reversed through the association with a pleasant game. The dream experience simultaneously allowed her to overcome the concern due to the burning experience, to avoid interrupting the funny play, and to recover her trust on burritos. Certainly, she was frightened by the dream, in the same way as she had being frightened while being burned. However, a basic point is that the evil tortilla only attempted to smother her.

Similar mechanisms can also be observed in dreams of famous people that have been reported in historical documents. For instance, at the age of 29, Charles Darwin dreamed of a corpse coming back to life after being executed (for a discussion about this dream, see Bulkeley, 2016). Clearly, a serious present concern in the dreamer's mind was due to the establishment's opposition to his theory. The dream experience represented the idea that the unjustly accused theory was able to come back to life. Thus, the dream experience presented a somehow ironically reversed form of the Christian dogma of Resurrection.

## Short Discussion

We now briefly consider the here proposed hypothesis from the points of view of the continuity theory, the plurality of dream meanings, and the respective role of conscious and unconscious items in dreams. The continuity theory states that close continuity exists between dreaming and waking life (see, e.g., Nielsen et al., 2004; Blagrove et al., 2011; Hobson and Schredl, 2011; Domhoff, 2017; Schredl, 2017). In particular, dream contents appear to be connected with important events in waking life (see, e.g., Perogamvros et al., 2013). The continuity theory has had many experimental confirmations and has often led to enlightening results about the process of dream building. In our case, the connection between recent and/or remote events in the real life is at the basis of the construction of the link patterns between sources, and the representative value of the dream experience is directly related to waking experiences. As we have observed in the above examples, elements of similarity between dream events and waking events are sometimes striking. The serial output offered by dreams simultaneously represent more than a single content, even though the contents can present contradictory aspects. This plurality of meanings maintains a trace of the distributed character of the dream-building system in the serial output. Certainly, a simple interpretation of a dream can often be obtained if a present concern is well known by the interpreter (who can be the dreamer himself or herself). However, because of the plurality of meanings of the dream experience, a simple interpretation, based on an arbitrary assumption of immediate clarity of the dream, is generally incomplete, if not astray, and often does not reveal the possible complexity of the feelings that contribute to the construction of the dream. The plausible association of distributed information with unconscious contents and of serial information with conscious contents, clearly expressed in the above cited review by Dehaene and Changeux (2011) is in agreement with the fact that the process of dream construction is essentially unconscious, while its output, the dream experience, is conscious, although in a way that is different from the consciousness of waking life. Of course, the unconscious character of the mechanism of dream construction from memory sources does not imply that these sources are unconscious as well: indeed, all of the present concerns and of the real-life events that were recognized in the above given examples were known by the dreamers. However, the phenomenon of the shift of the present concern shows that some contents, although being known by the dreamer, are not immediately recognized: this means that a significant form of involuntary reticence actually exists.

## Author Contributions

UB proposed the main idea of the paper after a discussion about the problem of seriality vs. parallelism in dreaming. All authors contributed to the above discussion. UB wrote the paper with many insights from PP and LS.

### Conflict of Interest

The authors declare that the research was conducted in the absence of any commercial or financial relationships that could be construed as a potential conflict of interest.
